# Metronomic Chemotherapy: A Systematic Review of the Literature and Clinical Experience

**DOI:** 10.1155/2019/5483791

**Published:** 2019-03-20

**Authors:** Cem Simsek, Ece Esin, Suayib Yalcin

**Affiliations:** ^1^Department of Internal Medicine, Hacettepe University, Ankara, Turkey; ^2^Department of Medical Oncology, A.Y. Ankara Training Hospital, Ankara, Turkey; ^3^Department of Medical Oncology, Hacettepe University, Ankara, Turkey

## Abstract

Metronomic chemotherapy, continuous and dose-dense administration of chemotherapeutic drugs with lowered doses, is being evaluated for substituting, augmenting, or appending conventional maximum tolerated dose regimens, with preclinical and clinical studies for the past few decades. To date, the principle mechanisms of its action include impeding tumoral angiogenesis and modulation of hosts' immune system, affecting directly tumor cells, their progenitors, and neighboring stromal cells. Its better toxicity profile, lower cost, and easier use are main advantages over conventional therapies. The evidence of metronomic chemotherapy for personalized medicine is growing, starting with unfit elderly patients and also for palliative treatment. The literature reviewed in this article mainly demonstrates that metronomic chemotherapy is advantageous for selected patients and for certain types of malignancies, which make it a promising therapeutic approach for filling in the gaps. More clinical studies are needed to establish a solidified role for metronomic chemotherapy with other treatment models in modern cancer management.

## 1. Introduction

While our understanding about the biology of cancer and the interaction of malignant cell with their microenvironment has improved, the research revealed that, apart from the molecule administered per sé, the dose and scheme of administration are important for therapeutic efficacy [1]. The idea of metronomic chemotherapy, the term first used by Hanahan, was first revealed with this standpoint [2]. Preclinical and clinical studies have been investigating the use of metronomic therapy as an augmentation or as a substitute for conventional regimens [3]. However, there is still ongoing debate about the current role of metronomic regimens in the treatment of cancer. The purpose of this systematic review is to reevaluate the position of metronomic chemotherapy in modern cancer management and make a projection about the future role in the treatment of malignancies.

## 2. Materials and Methods

Literature searches of PubMed (2005 to November 2017), ISIS (2005 to 2017), American Society of Clinical Oncology (ASCO) Annual Meetings (2005 to 2017), and European Society for Medical Oncology (ESMO) Congresses (2005 to 2017) were performed. The reviewing process was done in compliance with the Preferred Reporting Items for Systematic Reviews and Meta-Analyses statement (PRISMA) [4]. Articles were also screened manually and related citations were included into the systematic review to increase the sensitivity.

Studies conducted in adult patients in English language, published in peer-reviewed journals as phase II or III randomized controlled trials (RCTs) comparing continuous chemotherapy to an intermittent strategy of chemotherapy, with or without maintenance therapy including at least one of the outcomes of interest, were included. The ASCO and ESMO meeting abstracts as well as systematic reviews and meta-analyses were also accepted for inclusion.

## 3. Results

### 3.1. Literature Search Results

Entire literature search retrieved 5285 results. 1263 were regarded as potentially relevant and fully reviewed. 266 of them were retained in the study. Twelve abstracts from ASCO meeting abstracts were retrieved and 4 of them were retained. Two ESMO meeting abstracts were also included.

### 3.2. The Definition of Metronomic Therapy

The term “metronomic chemotherapy” (MTC) is currently used for frequent and regular administration of lower doses of chemotherapeutic drugs with minimal drug free time intervals, or simply “lower doses, longer times”, in order to establish a prolonged and lower albeit an active range of plasma concentration enabling a favorable side-effect profile [5]. In the opposite way, conventional regimens are used as maximum tolerated dose (MTD), in which relatively high doses are given with 2-3-week intervals [6].

### 3.3. The Mechanisms of Metronomic Action

**Figure 1:**
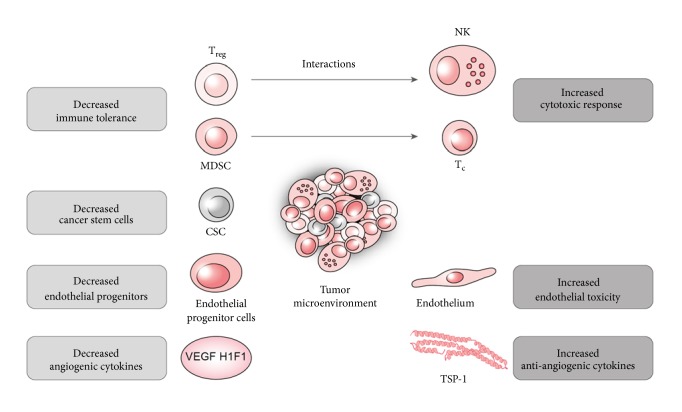
Proposed mechanisms for actions of metronomic chemotherapy regimens.

The preliminary role of MTC is derived from its antiangiogenic mode of action (Figure 1). This mode of action is shared by two classes of therapies, metronomic chemotherapy and anti-VEGF monoclonal antibodies. However, they have several different aspects. The antiangiogenic drugs directly impair the action of vascular endothelial growth factor (VEGF) and the metronomic chemotherapy disables the cells enrolled in the angiogenic mechanisms, suggesting that the tumoral endothelial cells could be a better target to overcome the drug resistance (Tan et al.). Tumor endothelial cells (TEC) are distinguishable from typical endothelial cells as they have different characteristics of proliferation, migration, genetic outline, and discrete reactions to growth factors. TEC is a major target for the antiangiogenetic action of MTC, which is selectively sensitive to metronomic administration of certain types of drugs [7–12]. The endothelial progenitor cell (EPC) is another major player in tumor vasculogenesis, which is another target of MTC [13, 14]. Apart from toxicity to endothelial cells, MTC also induces antiangiogenic protein Thrombospondin-1, inhibits angiogenic HIF-1*α*, and decreases circulating VEGF levels [15–18].

Secondly, as the tumor transforms its surroundings to create a viable microenvironment, it modifies the hosts' immune system and establishes a conciliatory climate. Restoring and enhancing the antitumor immune response is another aspect of metronomic regimens (Figure 1) [19]. Regulatory T cell (Treg) as one of the key players “locks down” cytotoxic response and thus maintains the tumor immune-tolerance [20]. MTC selectively depletes Treg and thus restores the natural killer (NK) and T cell functions [21, 22]. Repression of the myeloid derived stem cell, another immune suppressor, was also demonstrated in clinical trials [23, 24]. Besides, MTC enhances the cytotoxic response by inducing the antigen presenting dendritic cell maturation, augmenting its function and increasing tumorigenic antigen presentation that assembles the immunogenic cancer death. Immunogenic cancer death is the process in which the dendritic cell recognizes the immune-adjuvant damage associated molecular patterns (DAMPs) like HGMB-1, calreticulin, and ATP and complements the cytotoxic cell death [25–33]. With its immune modulatory aspects, MTC was shown to increase potency of other immune stimulatory modalities like vaccines [33, 34].

Another resident in the cancer microenvironment are cancer stem cells, which were discovered to take part in cellular dedifferentiation, tumoral heterogeneity, invasion, metastasis, and drug resistance [35, 36]. MTC was shown to decrease the number of triple positive CD133+/CD44+/CD24+ and CD44+ stem cells; however, its therapeutic implication has not been well defined yet [37, 38]. Heterogeneity of cancer cells can stem from the positive selection of resistant cancer clones after chemotherapy. With this perspective, the “chemo-switch” protocol, studied by Pietars et al, to control the tumoral stromal cells was also suggested to surpass this evolutionary process [39, 40].

Tumoral dormancy arises when proliferation of cancer cells is countervailed by their apoptosis [41]. The major factors hypothesized to maintain this balance are influence of microenvironment, impediment vascularization (angiogenic dormancy), and immune surveillance [42]. There are plentiful data that demonstrate that MTC particularly induces angiogenic dormancy by upregulating and downregulating antiangiogenic factors such as TSP-1 and proangiogenic factors such as VEGF, respectively [43].

Another demonstrated mechanism of action is selective modulation of certain gene and protein functions in tumor cells, which can be used as a means of an antiproliferative effect on tumor cells or to sensitize the tumor to certain chemotherapy actions [44].

With its aforementioned interaction of these factors, MTC has been deduced to have a role in induction of tumor dormancy (Figure 1) [45]. Additionally, MTC has been shown to decrease metastasis; however, the mechanism of this effect has not been established [46, 47].

### 3.4. Clinical Experience in Breast Cancer

Breast cancer is one of the broadest tracks for the “metronomic march.” The growing number of patients with incurable metastatic disease who need palliative treatment, the cumulative toxicity of cytotoxic therapy, and lastly the economic burden led a concerted effort to find alternatives for conventional regimens including taxanes, anthracyclines, pyrimidine antimetabolites, and tubulin inhibitors [48].

Cyclophosphamide (CP) and methotrexate (MTX) were on the top of the list to be tested in metronomic scheme in breast cancer [49–51]. The earlier studies investigated the oral metronomic use of CP and MTX together (CM) in pretreated metastatic breast cancer patients (Table 1). Colleoni tested the combination of CP 50 mg daily with MTX 2.5 mg twice daily 2 days per week, obtaining objective response rate (ORR) of 20.9% and a clinical benefit [18] (at least 24 weeks of objective response and stable disease) of 31.7% [49]. In the long-term follow-up, those patients yielded a 15.7% prolonged clinical benefit (longer than 12 months) [52]. Miscoria et al. and Gebbia et al. showed similar disease control and tolerable toxicity profiles in trials tested for CP alone or in combination with MTX [53, 54]. Metronomic CP and MTD liposomal doxorubicin combination were evaluated in metastatic patients; CB was 75% with a median OS of 6.4 months [55].

A more recent study in pretreated metastatic breast cancer combination of metronomic CP with MTX was tested; medians of PFS and OS were 5 and 7 months, respectively. Out of 48 patients, 1 patient had complete response [56]. In another study with metastatic hormone receptor positive patients, metronomic combination of CP with vinorelbine and capecitabine was evaluated for both naïve and pretreated patients. Naïve patients had a TTP of 25.1 months, while pretreated patients' median TTP was 11.2 months [57]. Zhang et al. evaluated addition of metronomic CP to docetaxel in non-triple-negative patients as a first-line treatment compared to docetaxel alone. There were no differences between groups with respect to the ORR, PFS, and OS; the authors concluded that the combination was not effective in this setting [58].

CP and MTX have a diverse combination schedules with targeted agents. Bevacizumab and trastuzumab (in HER-2 positive patients) with CM combination were tested by Garcai-Sanex et al. in a population of taxane and anthracycline refractory patients. An overall survival (OS) of 13,6 months was achieved [59]. The highest clinical benefit rate (68%) was reported with the combination of CP with bevacizumab and capecitabine in pretreated breast cancer patients. Vandetanib was also integrated with CM based metronomic therapy in a phase I dose escalation study. Adverse events resulted in loss of chemotherapy adherence in 1/3 of patients yielding a 10% partial response of the remaining 20 patients [60]. Perraud et al. experimented CM plus a selective cyclooxygenase-2 inhibitor celecoxib in 15 patients; clinical benefit (CB) rate was 46.7% with no serious toxicities [61]. Aurilio et al. used CM with fulvestrant, which resulted with prolonged CB [62].

The aim of metronomic treatment is not solely palliative treatment; neoadjuvant setting is an active area of investigation. Dellapasqua et al. reported the results of CP plus liposomal doxorubicin (PLD) as an initial therapy in locally advanced breast cancer patients; the rate of breast conserving surgery was found to be 44.8%. Additionally, 62.1% of patients were reported to have a PR, importantly without grade 4 toxicity [63]. An immunogenic mechanism was also explored with CM and an immunogenic vaccine; ORR was 23.8% [64].

A metronomic based chemohormonal scheme, CP plus megestrol acetate was used in 29 patients, ORR was 31.0%, and CBR was not reported [65]. An all oral scheme of CP (65 mg/m^2^ daily on days 1-14) plus capecitabine (1,000 mg/m^2^ twice daily on days 1-14 repeating every 28 days) treatment was evaluated in 68 pretreated patients. After median follow-up of 26 months, a CB was 53%; grade 4 toxicity occurred in 5% of patients [66]. Another CP (33 mg/m^2^ twice daily, days 1-14) and capecitabine (capecitabine 828 mg/m^2^ twice daily) treatment repeating every three weeks by Yoshimoto et al. resulted in a clinical benefit of 57.8% without any grade 4 toxicity [67].

As an extensively experienced drug for about 5 decades, 5-FU is still being used for breast cancer. With the discovery of capecitabine, an oral prodrug of 5-FU, thymidylate synthase inhibitors have been extensively researched for metronomic use [68]. Oral capecitabine (828 mg/m^2^ twice daily) with weekly paclitaxel was evaluated by Taguchi et al. PFS and OS were reported to be 8.3 months and 22.9 months, respectively [69]. First phase III study was published by Watanabe et al. comparing the adjuvant activity of oral uracil-tegafur with conventional CMF (cyclophosphamide, methotrexate, and fluorouracil) in node negative high-risk breast cancer patients. With 733 patients and 6.2 years of median follow-up, relapse free survival and overall survival rates of two combinations were similar, and uracil-tegafur group expressed a better quality of life [70]. Capecitabine was also combined with an oral tubulin inhibitor, vinorelbine. Cazzaniga et al. used the metronomic combination of vinorelbine with capecitabine with CB of 58% in 34 patients [71–73]. Same team conducted another study using the same combination; CB was 45.7% and 51.1% in first-line and second-line therapies, respectively. [74] Young et al. investigated the capecitabine with weekly docetaxel to low dose of taxane therapy to induce thymidine phosphorylase expression with addition of daily celecoxib; a 42% of CB was observed with median time of disease progression (TTP) of 3.6 months [75]. In hormone receptor positive tumors, conventional scheme of fulvestrant was added to capecitabine in 41 patients; CB of 58.1% was obtained with 14.9 months' median PFS [76]. A newer study by Otsuka et al. demonstrated an OR rate of 47% with metronomic tegafur-gimeracil-oteracil and MTD irinotecan [77].

Triple negative breast cancer (TNBC) is another area where we have a significant shortage of viable treatment strategies; metronomic chemotherapy can be employed in multiple settings. An elegant preclinical study demonstrated the action of metronomic chemotherapy in TNBC. In this study, metronomic topotecan was combined with pazopanib in an orthotopic metastatic breast cancer model to evaluate its potential mechanism of actions and the therapeutic efficacy. The combination was shown to modulate angiogenesis, drug resistance, apoptosis, and proliferation and subsequently prolonged the survival [78]. For neoadjuvant regimens, metronomic CP was recruited with weekly paclitaxel after epidoxorubicin-cisplatin-fluorouracil (ECF). Pathological response rates were evaluated; posttreatment Ki-67 was found to be decreased by 41% and 91% of the patients had complete pathological response [79]. Metronomic chemotherapy has also been evaluated for first-line therapy in metastatic TNBC; a multicenter phase III study compared the toxicity and efficacy of bevacizumab combined with metronomic CP versus bevacizumab with paclitaxel; there were no differences in ORR or PFS. A novel poly-ADP-ribose-polymerase inhibitor drug veliparib was evaluated in BRCA associated metastatic pretreated TNBC; objective response occurred in 43% of BRCA associated patients and 11% of BRCA negative/unknown patients [80]. Metronomic chemotherapy was also evaluated as maintenance therapy in TNBC patients. In a prospective controlled study with 158 stage II-III TNBC patients, group treated with additional maintenance metronomic after adjuvant FEC-100 and docetaxel was compared to control group without maintenance therapy; metronomic group's DFS and OS were 28 and 37 months, respectively, compared to control groups' DFS of 24 months and OS of 29 months [81]. For advanced pretreated TNBC, Viale et al. tested the combination of metronomic CP with cisplatin yielding a 23.3% clinical benefit at 6 months after treatment [82]. A different paper evaluated postadjuvant (FEC100 + radiotherapy) metronomic capecitabine of 6 months; mean disease free survival was 42.4 months [83].

Antiangiogenic action of microtubule inhibitors is important as a means to metronomic therapy. Oral form of vinorelbine has been experimented in 34 elderly metastatic breast cancer patients; an OR of 38% was reported [84]. The same author used temozolomide during whole-brain radiotherapy and metronomic vinorelbine afterwards in 36 patients with cerebral metastasis; OR was 52%. [85]. Vinorelbine was added to bevacizumab in a trial by Saloustras et al. but study was closed prematurely due to lack of efficacy (OR was 7.7%) [86]. Another study with an alternative on and off metronomic regimen of vinorelbine was dosed every other day for 4 years with a cumulative dose of 30 mgs; a 50% CB was reported, without grade 3 or 4 toxicity [87].

Oral etoposide is a well-tolerated and effective drug for metastatic breast cancer. Two decades ago, Calvert et al. used oral etoposide of 50 mg/m2 for first 14 days of 28-day cycles in 38 pretreated metastatic breast cancer patients. Eight of the patients had a partial response with median TTP of 16 weeks [88]. Bontenbal also used etoposide with 50 mg/m2 orally for first 21 days of 28 days in 27 pretreated metastatic breast cancer patients, achieving a CBR of 43% [89]. Another two-phase II trial used the same scheme in 43 and 18 pretreated metastatic breast cancer patients; ORR of 35% and PR of 21% were reported, respectively [90, 91]. The same regimen was used as a first-line drug in metastatic patients; one CR and five PR were obtained [92]. In a recent multicenter phase II trial, oral etoposide of 60 mg/m2 in first 10 days of 21-day cycles was used in 75 patients. A CB of 21.3% was achieved with median PFS of 4.5 months [93].

Metastatic breast cancer is a diverse and heterogenous disease with specific targets in which stepwise and sequential treatment can add survival benefit at the end. Hence, metronomic treatments are well known and extensively studied for these types of tumors. In metastatic setting, for hormone-receptor expressing tumor types, weekly paclitaxel, oral vinorelbine, capecitabine, and ixabepilone have proven efficacy with different side effect profiles. In triple negative tumor type, capecitabine is now a standard approach after neoadjuvant setting for patients with residual disease. In heavily pretreated patients, for palliative purpose, oral CYP and etoposide were used either as single agents or alternatively.

### 3.5. Clinical Experience in Castration-Resistant Prostate Cancer

Castration-resistant prostate cancer (CRPC) is an area in which there are significant gaps in therapy with current strategies; for now progressive disease is inescapable eventually. But frailty of patients makes the management of this disease more difficult. There has been a comprehensive research for treatment CRPC. Hence, the research continues for possible treatment options for docetaxel-resistant tumors (i.e., androgen synthesis inhibitors, specific or nonspecific immunotherapy, mitoxantrone, and targeted therapy), and when the frailty of the patients is taken into consideration, metronomic therapies were repeatedly tested (Table 2) [94].

For CRPC, metronomic CP was an early drug to be tested and, combined or alone, is still a favored choice. A study investigating CP in CRPC was by Raghavan in 1993; of 30 HRPC patients, 18 had a CB and improvement of the symptoms [95]. After a decade, Nicolini et al. used CP in eight metastatic HRPC patients; a CB of 62.5% and a greater than 50% PSA response in 2 patients were reported [96]. Of 80 other patients in whom CP was tested, rate of response was 34.5% including both objective and prostate specific antigen response [97]. Glode et al. tested CP with corticosteroids, as both drugs have been used for CRPC and both were shown to have antiangiogenic properties. Of 34 patients, 26% experienced disease progression and 6% were found to have a <50% decrease in PSA; 29% of patients were found to have a greater than or equal to 80% and 39% were found to have a 50-79% reduction in PSA [98]. Another study with 18 patients and a shorter follow-up of 12 weeks reported a decrease in PSA of greater than 50% in 23.5% and stable disease corresponding to a PSA response of less than 50% was seen in 29% of the patients [99]. In a retrospective analysis of 40 patients, PSA response rate was achieved in 20.0% of patients [100]. A retrospective analysis of CP plus prednisolone regimen reported a more than 50% PSA decrease in 26%of patients [101]. Additionally, in a study of 24 patients, the median PSA progression-free survival was 5.0 months and a PSA decrease of 50% was observed in 8 patients (33.3%) [102]. In a multicenter retrospective study with 48 patients pretreated with docetaxel and another drug, efficacy of metronomic CPA was retrospectively evaluated. 14% of patients had a biochemical response (PSA decrease greater than 50%); median PFS and OS were reported as 3,5 and 6,9 months, respectively [103]. Another study evaluating biochemical response for 18 patients with median 2 months of metronomic CPA exposure reported a biochemical response of 44% [104]. Fea et al. evaluated the efficacy of metronomic CPA with ongoing LHRH agonist therapy until disease progression or toxicity in pretreated patients; PSA response rate was 16% without any grade 3 or 4 toxicities [105].

There are other possible effective combinations of cyclophosphamide in pretreated CRPC. In a phase II trial combining DES with CP and corticosteroids, with reference to prior shown success of DES in hormone refractory disease, 15 (42%) of 36 patients had a >50% PSA response and the overall median survival was 16.4 months [106]. Celecoxib was also used with CP as it has been shown to have antiangiogenic action [107]. In consecutive elderly 29 patients, 13 (45%) had a confirmed PSA decrease of 50% or greater [108]. Another study combining celecoxib with CP, by Orlandi et al., 14 (32%) showed a PSA >50% decrease; the study also investigated the pharmacogenetics of VEGF-A and showed that a genotype of VEGF-A has impacts on FPS. In 28 advanced CRPC patients, Fontana et al. studied CP 50 mg daily with Celecoxib 200 mg twice daily and dexamethasone 1 mg daily. 32% of the patients had a PSA response. Median OS and PFS were 3 months and 21 months, respectively [109]. In another phase II trial, CP 50 mg daily was combined with MTX 2.4 mg twice a week. PSA response was observed in 25% of the patients [110, 111]. Dexamethasone combination with celecoxib and metronomic CPA was tested by another group for pretreated CRPC; reported PSA response was 39% with a median OS of 13,3 months for 22 patients [112]. CP and MTX joined with celecoxib were evaluated in another phase II trial, but the progression rate was 65.7% and there were no objective responses [113]. In a phase I trial, adding thalidomide to CP, 10 out of 13 patients (76.9%) had a progression of PSA > 25%, with 2 (15%) having a >50 reduction [114]. CP, corticosteroid, capecitabine, and thalidomide were evaluated in 8 patients for a median time of 6 months; overall survival was 19.5 months [115]. Another study by Bracarda et al. used estramustine and CP in docetaxel naive patients; the 50% reduction was seen in 14 (43.7%) of 32 patients [116]. Nishimura et al. added tegafur to the estramustine and CP combination; 12 (57.1%) of 21 patients showed a PSA decline of 50% or greater [117]. Hatano et al. retrospectively evaluated oral UFT and CP with dexamethasone in 57 patients. 63% of PSA response was achieved; in the PSA responder group, median time to progression was 13.3 months [118]. Derosa et al. combined first- and second-line drugs in metastatic chemotherapy-naive CRPC patients and used CP and prednisone together with docetaxel; of 41 patients, 87% were free of progression at 6 months, and a decrease in PSA 50% was observed in 82%. No grade 4 toxicities were reported, with grade 3 toxicities being neutropenia (5%), thrombocytopenia, diarrhea, and stomatitis (2.5%) [119]. Another modified scheme consisting of ketoconazole in combination with estramustine, cyclophosphamide, or etoposide administered on alternate weeks, suggested by Jellvert et al, achieved a 59% decrease in PSA >50% [120]. In another multicenter trial with oral etoposide and estramustine 15 mg/kg daily in 55 patients, 22% of PSA response was reported [121]. Oral dexamethasone 0.5 mg daily alone was evaluated in 102 castration-resistant patients with 49% achieving a PSA response [122]. Daily oral CP 100 mg with 50 mg etoposide (14/21 days) was evaluated in 20 hormone refractory patients; an OR of 35% was reported [123]. In a retrospective evaluation of oral dexamethasone regimen in 99 patients, 40.4% of PSA response was found [124]. Intravenous vinorelbine 25 mg/m^2^ weekly for first 12 weeks and biweekly afterwards was used with low dose oral prednisone in 14 patients, with a PSA response in 29% [125]. Oral metronomic vinorelbine was also evaluated with serum markers of tumor response and activity. PSA response was 61%, and a decrease in VEGF and TSP-1 was observed in responders [126]. Vinorelbine alone was compared to weekly docetaxel in frail CRPC patients for efficacy, tolerability, toxicity, and compliance. Efficacy and tolerability of the two regimens were found to be similar in elderly unfit patients [127]. In another phase I/II trial, CP 50 mg daily with lenalidomide 25 mg daily in first 21 days repeating every four weeks was tried in 6 patients with PSA reduction in 31.7%, with improved markers of neovascularization [128]. Oral etoposide 25 mg twice daily with oral prednisone twice daily was administered for 21 of 28-day cycles; 41% of biochemical response was achieved with a PFS of 5.9 months [129].

One study has evaluated the metronomic therapy in nonmetastatic prostate cancer. In this prospective single-arm study, metronomic CPA was administered for 6 months to patients with only biochemical recurrence after curative local therapy before androgen deprivation. 38 patients were enrolled; 37% of patients had a PSA stabilization, and 58% had PSA progression [130].

In castration-resistant prostate cancer, with the invention of new class antitestosterone drugs (enzalutamide and abiraterone), the role for CYP, etoposide, estramustine, and ketoconazole diminished. On the other hand, in a limited number of patients who were progressed on standard approaches and still in need for treatment, oral etoposide and ketoconazole may have a role. Of note, dexamethasone may have both tumor-static effects and antiangiogenic effect in addition to blocking androgen synthesis for CRPC patients.

### 3.6. Clinical Experience in Ovarian Cancer

High recurrence rates even after achievement of complete response to standard surgical debulking and platinum-based combination therapy make ovarian cancer a challenging entity for clinicians; thus newer therapeutic approaches have been under investigation. As angiogenesis plays a prominent role in pathogenesis of ovarian cancer, metronomic chemotherapy with other antiangiogenetic agents has been a distinguished area for research [131] for both first-line, maintenance and salvage therapy (Table 3).

Bevacizumab as an antiangiogenic molecule and its synergism with metronomic chemotherapy were tested for ovarian cancer [132]. Bevacizumab was incorporated into standard first-line therapy in a phase III study by Burger et al. fortifying the position of bevacizumab, comparing patients having carboplatin paclitaxel in three groups. The First one was without bevacizumab and the second and third groups were with initial and throughout bevacizumab, respectively. It was reported that bevacizumab prolonged the median PFS by about 4 months with a median time of 14.1 months versus control groups' 10.3 months [133].

Two studies evaluated metronomic regimens for maintenance therapy. In a nonrandomized study enrolling ECOG 0-1 patients with complete response to standard first-line therapy, metronomic regimen of CPA and MTX was compared to observation alone. Maintenance arm had a longer PFS of 18 months versus 15 months of observation arm without any grade 3 or 4 toxicity. Another retrospective study evaluated a group of patients who were administered a metronomic regimen for neoadjuvant therapy and also 6 months as a maintenance after adjuvant standard therapy. Metronomic neoadjuvant plus metronomic maintenance group was reported to have a prolonged DFS of 3 months without increased toxicity profile [134].

Neoadjuvant chemotherapy is standard before maximal debulking surgery; nevertheless there is an important fraction of patient who are not suitable for effective but highly toxic platinum-based regimens. Dessai et al. used paclitaxel and carboplatin with an alternative weekly metronomic scheme (80 mg/m2 / AUC-2) in patients regarded as unsuitable for standard 3-week regimen. The response to the neoadjuvant chemotherapy was 100%; two grade 3-4 toxicities were reported [135].

Although response rates of ovarian cancer to platinum regimens are considerably high, platinum resistance is inevitable; thus developing treatment strategies in case of platinum-refractoriness is an essential area for research. Oral etoposide of 50 mg/m^2^ for twenty days repeated every 28 days was used in 18 pretreated patients with 1 patient having a partial response of 11 months [136]. In another multicenter retrospective study using the same dose of oral etoposide for 14 days of 21-day cycles regimen in 51 platinum-resistant patients, PFS of 3.9 months and OS of 16.4 months were achieved [137]. As cyclophosphamide is a part of conventional treatment of ovarian cancer and it is favorable for metronomic regimen, Samaritani et al. tried metronomic cyclophosphamide in a 17-year-old female with advanced ovarian cancer with PFS of 65 months. [138] Another study experiencing oral CP alone as salvage therapy in platinum-sensitive heterogeneous patients reported median PFS for 4 months and median OS of 13 months [139]. Metronomic doses of CPA plus temozolomide were studied in 55 platinum-refractory patients; an ORR of 44.4% was achieved with a median PFS of 5.9 months and a median OS of 10.1 months [140]. Again in platinum-refractory patients, CPA was combined with antiangiogenic pazopanib with metronomic dosing scheme with median PFS and OS being 8,3 and 24.9 months, respectively [141].

CP and bevacizumab were also tested for pretreated platinum-resistant ovarian cancer. Two studies by Sanches et al. and Garcia et al. combined CP 50 mg/day with bevacizumab 10 mg/kg intravenously every 2 weeks; the PFS was 4.5 months and 7 months and OS was 7 and 17 months, respectively [142, 143]. Barber et al. also reported an OS of 20 months in 66 patients [144]. Jurado et al. retrospectively evaluated CP and bevacizumab; median progression time was 5.5 months [145]. In another study using the same combination with identical dosage in heavily pretreated patients with a median previous chemotherapy number of 8, a total response of 53.3% was reported [146]. Bevacizumab was also integrated with other conventional regimens. Topotecan in cycles of 1, 8, and 15 days of 28-day administrations with biweekly bevacizumab resulted in PFS of 7.8 months and OS of 16.6 months [147]. Pujade-Lauraine et al. experimented bevacizumab with three different combinations: pegylated liposomal doxorubicin, weekly paclitaxel, and topotecan; the median PFS was 3.4 months with chemotherapy alone versus 6.7 months with bevacizumab [148]. Weekly administered ixabepilone was retrospectively evaluated with or without bevacizumab for 24 uterine and 36 ovarian, primary peritoneal and fallopian tube cancers. For uterine cancers, addition of bevacizumab significantly increased both PFS (3.0 months versus 6.5 months) and OS (4.2 months versus 9.6 months); it was reported that similar results were estimated for ovarian cancer [149]. A meta-analysis evaluating the role of bevacizumab concluded that there is an advantage of PFS and OS when chemotherapy was combined with bevacizumab and increased risk of non-CNS bleeding, hypertension, gastrointestinal perforation, thromboembolism, and proteinuria [150]. The tyrosine kinase inhibitor sorafenib was also investigated for metronomic efficacy with topotecan; grade 3-4 toxicities of leukopenia/neutropenia (23%), thrombocytopenia (17%), and anemia (10%) had occurred. Of 16 patients, PR was reported in 5 (16.7%) and SD was reported in 14 (46.7%) [151]. Celecoxib was joined with carboplatin by Legge et al. Median PFS and OS were 5 and 13 months, respectively [152]. A cohort comparing metronomic thalidomide versus tamoxifen in only biochemically recurrent ovarian cancer was closed as interim analysis and it was shown that thalidomide was not more effective in reducing the recurrence rate relative to tamoxifen and higher toxicity rate [153]. Another study evaluating metronomic thalidomide versus single-agent chemotherapy in recurrent ovarian cancer and primary peritoneal cancer showed no significant difference [154]. Thalidomide with standard topotecan resulted in 2 months' increase in PFS in a different phase II trial [155]. Noronha et al. evaluated weekly paclitaxel 80 of mg/m2 in platinum refractory and platinum ineligible 37 non-small cell lung cancer patients; the response rate was reported as 35%, with median PFS of 4 months [156]. Temozolomide twice daily for fourteen days repeated every three weeks was combined with daily CP in 54 patients. There were grade 3 and grade 4 toxicities which were mostly hematologic. Overall response was 44.4%; median PFS was 5.9 months [140].

In conclusion, metastatic epithelial ovarian cancer is a long-standing malignancy with a need for further treatment options. In heavily pretreated patients, oral metronomic CYP has a role. Topotecan is also an effective agent for platinum-resistant patients as a single agent. Bevacizumab is an approved agent in both platinum-sensitive relapsed and resistant ovarian cancer patients in induction and maintenance period. There is a growing interest for further studies in metronomic, angiogenesis-targeted treatment approaches in this tumor type.

### 3.7. Clinical Experience in Glial Cancer

GBM has long been treated with temozolomide (TMZ) but the results are dissatisfying. The metronomic approach to TMZ and GBM has been beheld since the inhibition of O6-methylguanin-DNA-methltransferase (MGMT) by prolonged TMZ exposition [157].

A trial with metronomic therapy of alternating etoposide-cyclophosphamide with daily thalidomide and celecoxib did not increase survival rates (Table 5) [158]. Several clinical trials experimented the metronomic TMZ. Clarke et al. compared six adjuvant cycles of either MTD (150 mg/m^2^, days 1 to 7 and 15 to 21) or metronomic (50 mg/m^2^ daily) TMZ following standard radiotherapy and daily temozolomide in 85 patients. For MTD and LDM regimens, the 1-year survival rates were 80% and 69% and the median OS was 7.1 months and 15.1 months, respectively [159]. Another study by Kong et al. showed that metronomic TMZ can be effective for the patients refractory to standard cyclic treatment, with 15 patients, with 50 mg/m^2^ daily TMZ; 6-month PFS was 32.5 months and 6-month OS was 56.0% [160]; this rescue approach was also supported by another study. Metronomic TMZ was experimented in similar doses until progression in highly pretreated patients including ones with bevacizumab exposure; 6-month PFS was 19% [161]. Again, another study using 8 weeks of metronomic TMZ in relapsed GBM patients yielded a median OS of 6 months with a 6-month PFS of 20%. Authors also evaluated the micro vessel density in patients who needed reoperation after maintenance therapy and demonstrated a decrement [162].

Bevacizumab refractory patients were reported to have a worse response [163]. A published meta-analysis compared metronomic and standard TMZ regimens; although 6-month OS did not have a significant difference, PFS was detected to be significantly higher in metronomic schedules [164]. TMZ was also combined with other antiangiogenic agents like COX-2 inhibitors; Tuettenberg et al. showed that the metronomic TMZ and rofecoxib combination showed antiangiogenic action [165]. A similar study with TMZ and celecoxib in refractory GBM patients resulted in a PFS of 6 months in 43% of patients [166]. Another current approach for treatment of GBM with bevacizumab was also evaluated by Reardon et al. using bevacizumab with metronomic etoposide for recurrent GBM; authors reported similar activity but increased toxicity [167]. Same authors also tried metronomic etoposide or temozolomide administered with bevacizumab for bevacizumab recurrent GBM but the trial was closed at the first interim due to lack of activity [168]. Twenty-three high-grade glioma patients were administered bevacizumab (1 mg/kg every three weeks) and TMZ (50 mg/m2 daily) until clinical or radiological progression; 6 months of PFS were reported to be lower with respect to other bevacizumab-including regimens [169]. With respect to the results with bevacizumab, Zustovich et al. tried another tyrosine kinase inhibitor, sorafenib, twice daily with metronomic TMZ; 6-month PFS was 26%, and median OS was 7.4 months [170].

In a highly aggressive and resistant tumor type, in glial tumors, well-established metronomic treatment modality includes temozolomide. The addition of bevacizumab has conflicting results; however, it is accepted that targeting angiogenesis may improve progression-free survival.

### 3.8. Clinical Experience in Renal Cell Cancer

Targeted therapies have become the standard of care for renal carcinoma. Targeting angiogenesis, a key metabolism in oncogenesis of these tumors has led to improving the survival rate of these patients. On the other hand, agent specific toxicities (quality of life deterioration, anorexia, weight loss, and fatigue) are major concerns in terms of treatment adherence. Besides, as the progression-free survival duration prolonged, the risk of treatment resistance increases.

When resistance to targeted therapies emerged, a possibility to increase the efficacy of targeted therapies with metronomic scheduling with reference to preclinical knowledge raised and generated further clinical studies [39]. An elegant preclinical study demonstrated the efficacy of metronomic regimens. Metronomic topotecan was combined with pazopanib and tested against human RCC cell lines. The combination induced and maintained dormancy in metastatic foci. Pazopanib was also shown to increase intracellular topotecan levels [171]. An early study was by Bellmunt et al. investigating six cycles of combination therapy MTD gemcitabine combined with metronomic capecitabine and sorafenib for 6 cycles and followed by metronomic sorafenib. The median PFS for these patients was 11.1 months. Of the 44 patients, a partial response was achieved in 20 patients, and stable disease was reported in 17 [172]. Another study used etoricoxib plus pioglitazone daily, with low-dose interferon three times a week and capecitabine twice daily orally for 4 days, every 3 weeks. Median OS and PFS for the total cohort were 26.9 and 7.2 months, respectively. Grade 4 toxicity was seen in 48.8% [173]. In a recent trial by Tupikowski et al. metronomic CP and interferon *α* combination resulted in a CB longer than 24 weeks, which was observed in 40% in 30 patients; median OS was 13.2 months [174].

In RCC, sunitinib, pazopanib, and axitinib and in limited patients sorafenib have antitumoral efficacy. Beyond tyrosine kinase inhibitors and new era drugs, for immunotherapies, there is no proven drug which has a metronomic action. Sunitinib in standard doses may be less tolerated in frail and elderly patients. In such cases, 50 mg sunitinib in 14 days on/7 days off schedule may be an option.

### 3.9. Clinical Experience in Lung Cancer

Lung cancer is the leading cause of cancer-related death; unfortunately it is mostly diagnosed at an advanced stage. According to patient and disease characteristics, palliative or curative treatments may be chosen. Regarding this point, metronomic chemotherapy has been a consideration especially for elderly and debilitated patients.

For non-small cell lung cancer, metronomic regimens were tested as both a first line in frail patients and as a salvage therapy. Oral etoposide is a widely experienced drug for salvage therapies. Pfeiffer et al. compared 100 mg twice daily oral etoposide with conventional intravenous regimen for a palliative treatment option for small cell lung cancer (SCLC). 1-year survival was 9.8% in etoposide group with OS of 4.8 months, which were reported to be inferior to intravenous cyclophosphamide and etoposide or cyclophosphamide doxorubicin and vincristine regimens [175].

In another trial with oral etoposide alternating doses of 100 mg in non-small cell lung cancer (NSCLC) patients, partial response and stable disease were 28% and 34%, respectively, with median TTP of 6 months and median OS of 9 months [176]. An all oral regimen including etoposide 50 mg/m^2^ with UFT and leucovorin was used in pretreated advanced NSCLC. Grade 3 neutropenia and thrombocytopenia were observed in 12% and 15% patients, respectively, with rarer grade 3 nonhematologic toxicities. 14% of stable disease and 28% of partial response were achieved with a median TTP of 3 months [177]. Another all oral regimen with etoposide with lomustine and cyclophosphamide was used in 71 pretreated SCLC patients; ORR of 38% and severe but rare hematologic toxicity were reported [178]. An earlier trial by Correale et al. with weekly cisplatin (30 mg/m^2^, days 1, 8, 14, and 28) oral etoposide (50 mg/m^2^, 1-21 of 28 days) showed increased efficacy; overall response rate was 45.2%, grade 3 leukopenia and anemia have been seen, and 3 of 31 patients died from pulmonary thromboembolism [179]. Goern et al. used 25 mg/m^2^ weekly docetaxel and 50 mg daily trofosfamide in 62 stage IV NSLC patients. Overall response was 19%; median OS was 9.6 months; with PFS of 2.9 months [180]. Same authors studied efficacy of cisplatin 30 mg/m^2^ days 1-3, with bevacizumab 5 mg/kg in day 3 and oral etoposide in days 1-15 repeating every 3 weeks (mPEBev regimen) in 45 stage III/IV non-small cell lung cancer. Patients achieving stable disease or objective response were given erlotinib until progression. A partial response was reported in 31 patients, with progression-free survival of 9.53 months [181]. Kontopodis et al. used metronomic vinorelbine in 46 pretreated patients with a response rate of 10.9%; median OS was 9.4 months; 23.9% of patients showed grade 3-4 neutropenia [182].

Brain metastasis is a frequent progression in NSCLC progression. Low dose TMZ of 75 mg/m^2^ for 21 days every four weeks concomitant with whole-brain radiotherapy was administered in patients with brain metastasis. 2 complete and 11 partial responses were reported in 27 patients [183]. TMZ was also used in a study including brain-metastatic and non-brain-metastatic patients, with the dose of 75 mg/m^2^, yielding median survival of 3.3 months; grade 3 and grade 4 toxicities were reported [184]. Another study evaluating metastatic patients treated with metronomic regimens concluded that addition of radiotherapy may have a synergistic effect on overall survival [185].

Oral vinorelbine is a studied drug for front line, regarding that it has already been established as a front-line MTCR. Two studies combined oral vinorelbine. The first one combined vinorelbine with cisplatin as first line for inoperable advanced NSCLC; PFS and OS were 4.2 months and 12.0 months, respectively [186]. The second study by Tan et al. used oral vinorelbine with three different doses of 30-60-90 mg/week with sorafenib; median PFS was 4.4 and median OS was 8.2 months; the study showed no statistically significant difference among the three different doses [187].

Metronomic chemotherapy has also been used for patients who are ineligible for standard treatment options. Sorio et al. used oral etoposide with 17 elderly patients with advanced NSCLC with 100 mg daily for first 14 days of 3- or 4-week cycles; median OS was 24 weeks with one partial response and six stable diseases [188]. Camerini et al. evaluated oral vinorelbine in 43 elderly chemotherapy-naive patients, with OS of 9 months [189]. Two other studies also tested vinorelbine for frail patients. One of the studies used oral vinorelbine (30 mg, 3 per week) for 35 chemotherapy-naïve patients, yielding an ORR of 26%, median PFS of 4 months, and a median OS of 7 months [190]. In another study applying the same regimen in a similar frail population, median PFS and OS were 2,5 and 5,5 months, respectively [191]. Other regimens were tested for frail, advanced NSLC patients. A metronomic regimen of paclitaxel and gemcitabine was tested with a combination of bevacizumab with additional markers of vascularization. In 39 advanced NSCLC patients, ORR was 56%, and median PFS rates at 6 and 12 months were 61% and 21%, respectively, with a median OS of 25.5 months [192]. As radiotherapy is a conventional option in elderly patients ineligible for cytotoxic therapy, a study evaluated the addition of metronomic regimens to radiotherapy; no significant clinical efficacy was observed [193].

Efficacy of metronomic regimens as a maintenance chemotherapy was also tested. Maintenance treatment with oral etoposide after first-line docetaxel and cisplatin treatment was evaluated in metastatic NSCLC patients, although no complete response was observed; median overall survival was 10 months, with 1-year survival of 41% [194]. Another trial evaluating oral etoposide as a maintenance treatment was conducted by Li et al. in SCLC patients who responded to etoposide cisplatin regimen. 31 of 54 etoposide cisplatin responsive patients were evaluated; median PFS was 9 months and OS was 14 months [195]. In a recent trial, oral etoposide was combined with bevacizumab as a maintenance therapy following cisplatin, etoposide, and bevacizumab; median PFS and OS were 7.8 and 13.2 months, respectively [196]. Correale et al. used oral vinorelbine for >70-year-old patients. ORR was 18.6% [197].

Metronomic chemotherapy was far less studied for small cell lung cancer (SCLC). One study evaluated the efficacy of more affordable weekly paclitaxel over standard MTC topotecan for the second-line treatment. Median PFS and OS were reported as 145 and 168 days, respectively [198].

In the palliative setting for heavily pretreated NSCLC patients, oral etoposide is the most studied metronomic agent which may have an efficacy. Similar approach can be accepted also for SCLC patients who are in need for symptom control and treatment beyond first- and second-line approaches. In the elderly, frail patients who are not candidates for standard approaches, oral metronomic etoposide and vinorelbine may be the options. In the first-line treatment of NSCLC, systemic bevacizumab has a role as an antiangiogenic agent in addition to chemotherapy.

### 3.10. Clinical Experience in Gastrointestinal Cancer

Antiangiogenesis had been the area of interest in gastrointestinal malignancies for decades. Bevacizumab is the antiangiogenic agent that is approved for metastatic colorectal cancer patients. In addition to bevacizumab, pharmacokinetics of well-known chemotherapeutic agent fluorouracil had been studied for better efficacy and decreased toxicity. Hence, lowered but prolonged doses of standard chemotherapy for gastrointestinal malignancies have been proposed for better efficacy, decreased toxicity, and targeting angiogenesis.

Pharmacodynamic and pharmacokinetic profiles of metronomic regimens in gastrointestinal cancer were evaluated using a combination metronomic regimen of UFT, CP, and celecoxib in pretreated cases. This study showed that the cases of higher 5-FU peak concentrations and area under the curves had a better treatment response, thus elegantly illustrating the relation between pharmacokinetic profile and clinical efficacy. Moreover, pharmacodynamic profile of the combination was delineated by measuring the plasma levels of pro- and antiangiogenic molecules. Patients with higher proangiogenic molecules despite chemotherapy had more progressive diseases which proposed an antiangiogenic activity of the regimen [199]. In another study, metronomic irinotecan was tested in pretreated cases with three different metronomic dosage regimens. The combination achieved a similar response as conventional third- or fourth-line chemotherapy without any significant toxicity. Antiangiogenic molecule Thrombospondin-1 was shown to decrease concomitantly with irinotecan infusion, supporting an antiangiogenic action of the regimen [200].

An earlier study regarding metronomic chemotherapy in gastric cancer by Colleoni et al. used oral etoposide 50 mg/m^2^ with intravenous fluorouracil for fourteen days of 28-day cycles in 28 gastric cancers; an overall response of 50% was achieved with a median TTP of 4.5 months and OS of 9.5 months [201]. In another phase II study by He et al. in 45 pretreated elderly patients, 1000 mg capecitabine was administered throughout days 1–28 every 5 weeks. Objective response rate was 20.9%. The median TTP was 3.6 months and median OS was 7.6 months. No grade 4 toxicity was observed [202]. Weekly paclitaxel with lower doses of 80 mg/m^2^ was retrospectively evaluated on patients with unresectable esophageal cancer. After a median of 11 cycles, 71% of 51 patients had improvement in dysphagia. Overall response was 49%, with median progression-free survival of 4.7 months [203]. A different study retrospectively evaluated the efficacy of metronomic capecitabine in pretreated upper gastrointestinal tract cancers including patients with esophagogastric and pancreaticobiliary tumors with 31% of patients achieving clinical benefit [204].

Metronomic regimens were also evaluated for the second-line treatment of CRC. A phase II trial to evaluate the efficacy of metronomic UFT, CPA, and etoposide for first-line therapy in metastatic or recurrent colorectal cancer patients reported an ORR of 70% and a median OS of 23,5 months [205]. In a study evaluating efficacy of addition of metronomic tegafur/uracil (UFT) to 5-FU and oxaliplatin in 28 pretreated metastatic CRC patients, yielded median OS was 13.4 months [206]. Metronomic UFT was combined with weekly 40 mg/m^2^ irinotecan in 49 stage IIIb and stage IV patients, yielding 5-year survival of 73% and 62%, respectively [207].

Metronomic regimens are again an inviting option for frail patients. A study by Romiti et al. retrospectively evaluated efficacy of metronomic capecitabine of 1500 mg daily in 86 frail patients. Overall disease control rate was 26% with a 2% partial response and 23% stable disease. 19% of patients were progress-free for 6 months, and the median OS was 8 months. No grade 4 toxicity was observed [208]. Another trial also with pretreated frail elderly patients with advanced colorectal cancer evaluated the efficacy and toxicity profile of a metronomic regimen of capecitabine (1000 mg twice daily), oxaliplatin (65 mg/m^2^), and bevacizumab (7.5 mg/m^2^). No grade 4 toxicity was observed; progress-free survival was 12.3 median, with 86.7% reaching six months [209]. Capecitabine was also used in a metronomic regimen of 1,5 g daily in frail, recurrent, pretreated colorectal cancer patients. Disease control rate was 26% with a median OS of 8 months [210]. Another study retrospectively evaluating metastatic colorectal cancer patients reported a median TTP of 6.3 moths and a tolerable toxicity profile [211].

Metronomic maintenance strategies for RAS mutant colorectal cancer were also tested. In a study, RAS mutated CRC was evaluated for the response to metronomic maintenance regimens. Patients were given one of four conventional regimens (capecitabine or 5-FU plus oxaliplatin or irinotecan); then nonprogressing ones were randomized with their KRAS mutational status. KRAS mutant ones were randomized to metronomic capecitabine or bevacizumab and KRAS wild types were randomized to bevacizumab alone or bevacizumab plus erlotinib. The addition of erlotinib in KRAS wild type patients did not significantly prolong survival [212]. For stage III colorectal carcinomas, efficacy of metronomic UFT was questioned in a retrospective study of 113 patients; prolonged 5-year OS of 86.6% was noted in maintenance group compared to control groups, 68.5% [213]. In CAIRO 3 study, a phase III study was planned to ascertain the efficacy of maintenance metronomic treatment with capecitabine plus bevacizumab after an induction treatment with six 3 weekly cycles of capecitabine, oxaliplatin, and bevacizumab (CAPOX-B). 558 previously untreated metastatic CRC patients were allocated into either the maintenance or the observation group on a 1:1 basis. Capecitabine 625 mg/m2 oral twice daily and bevacizumab 7.5 mg/m2 intravenously every 3 weeks were the maintenance treatment. During the follow-up, progressing patients in maintenance of observation groups were given their second CAPOX-B; nonprogressing ones were followed. With a median follow-up of 48 months, PFS was significantly 3.2 months longer in maintenance group, 8.5 months versus 11.7 months. It was reported that the global qualities of life are similar between the groups [214]. A following randomized study questioned the efficacy of bevacizumab alone or combined with metronomic CPA plus capecitabine in unresectable CRC patients; the combination did not improve PFS [215].

In the metronomic treatment of gastrointestinal malignancies, especially for mCRC, the role of capecitabine with/without bevacizumab has a definitively important role in the palliative setting and in maintenance therapy for patients who have a response on first-line treatment.

### 3.11. Clinical Experience in Hepatocellular Cancer

Advanced hepatocellular cancer (HCC) has a dismal prognosis. In the early stages when the patients are candidates for systemic treatment, options are scarce. Systemic adriamycin was the only chemotherapeutic agent that was accepted as standard first-line treatment for patients who are not eligible for transplant or local ablative therapies. More recently, sorafenib was recognized as a standard treatment. As HCC is usually concomitant with cirrhosis, a tolerable combination and/or maintenance treatment with less adverse events is required to improve the survival benefit of sorafenib (Table 4).

Brandi et al. tested metronomic capecitabine in a 69-year-old patient with advanced HCC with therapeutic success [216]. Following this study, same team experimented metronomic capecitabine in 90 patients, in whom 59 were chemotherapy-naive and 31 were resistant or intolerant to sorafenib. Median PFS of first cohort was 6.03 months and OS was 14.47 months. Second cohort achieved a median PFS of 3.27 months and a median OS of 9.77 months [217]. Granito et al. also retrospectively evaluated the efficacy and safety of metronomic capecitabine in 26 patients pretreated with sorafenib. Median treatment duration was 3.2 months, median TTP was 4 months, and OS was 8 months [218]. Another trial combined sorafenib with metronomic UFT as a first-line therapy; median PFS and OS were 3.7 months and 7.4 months, respectively. Hand foot skin reaction occurred in grade 3 in 9% of patients and was reported to be the major adverse event resulting in dose reduction [219]. A different study evaluated the efficacy of bevacizumab with doses of 5 mg/kg or 10 mg/kg every two weeks in 43 advanced HCC patients; 16-week disease control rate was 42%. Grade 3-4 side effects including asthenia and hemorrhage were reported [220]. Shao et al. used an alternative regimen of thalidomide and metronomic UFT and also got comparable results of median PFS of 0.9 months and a median OS of 4.6 months [221]. A novel use of metronomic chemotherapy was experimented in a Korean trial. In 30 HCC patients with portal vein thrombosis, an intrahepatic arterial metronomic infusion of epirubicin, cisplatin, and 5FU was performed. Six patients achieved a partial response and six other patients had stable disease. The median overall survival was 162 days [222]. Success of metronomic capecitabine versus observation alone was retrospectively assessed as a second-line treatment; median PFS of the prior group was 12.0 months, while the other groups had shorter median OS of 9.0 months; authors concluded a 46% reduction in death risk [223]. Another study retrospectively analyzed the success of metronomic protocol of 5-FU, cisplatin, and capecitabine via hepatic arterial infusion chemoport versus sorafenib treatment in advanced HCC patients with portal vein thrombosis. OS was 158 and 117 days, respectively, for the two groups [224].

In summary, HCC has a dismal prognosis and beyond first-line treatment has little impact on OS of these patients. In terms of metronomic treatment, beyond TKI, capecitabine might have a role in patients with higher Karnofsky performance scores.

### 3.12. Clinical Experience in Multiple Myeloma

MTD with autologous stem cell treatment for available patients is a curative regimen for most of the middle-to-high-risk patients. Nevertheless, the morbidity of bone marrow transplantation and the toxicity profile of the commonly used drugs narrow down the treatment options, especially for relapsed or refractory multiple myeloma.

Vasculogenesis is an important element in pathogenesis of multiple myeloma; thus employment of antiangiogenic drugs with metronomic schedules can be rational [225]. The subject has also a historical value: a still used drug, thalidomide, was discovered to be antiangiogenic and it was first experimented in multiple myeloma [226, 227]. Cyclophosphamide and thalidomide derivatives are widely used drugs with a metronomic regimen.

For relapsed or refractory multiple myeloma (RRMM), Suvannasankha et al. combined oral cyclophosphamide (50 mg two per day for 21 days), thalidomide (200 mg daily day), and prednisone with 28-day cycles. In 35 patients, CB was 85.8%, with 20% complete response, 5.7% near complete response, 13% partial response, and 22.9% stable disease. Grade 3 and grade 4 toxicities were reported; hematological ones were most common [228]. Further studies evaluated thalidomide combined with CP and prednisone. Reece et al. combined lenalidomide 25 mg on days 1 and 21, cyclophosphamide on 300 mg/m^2^ on days 1, 8, and 15, and prednisone 100 mg every other day in a cycle of 28 days. With a median follow-up of 28 months, ORR was 94% and median PFS was 161 months [229]. Zhuo et al. evaluated metronomic CP with corticosteroids as salvage therapy in comorbid and heavily pretreated patients, ORR was 66.7%, and PFS of respondent patients was not reached at the study [230]. Same author questioned the use of metronomic regimens in patients with heart failure who are not eligible for standard treatment protocols. In 54 relapsed or refractory MM patients who also had a severe heart failure (NYHA III/IV), continuous low dose CP and dexamethasone were administered, 63% clinical benefit was achieved, and PFS was reported to be 6 months [231]. Papanikolau et al. retrospectively evaluated 186 multiple myeloma patients who were administered a novel metronomic regimen of bortezomib, thalidomide, dexamethasone, doxorubicin, and cisplatin with or without rapamycin. For 186 patients, median age was 61, with median 14 pretreatments. Patients have had median 1 cycle of therapy; median OS was 11.2 months. Median PFS was 3.6 months for an overall response rate of 63% (117 of 186 patients). Toxicities related to therapy were reported to be not trustworthy as some of the patients have had a hematologic condition prior to treatment [232]. Regarding RRMM patients, a prospective phase 1/2 study was conducted for the effectiveness of metronomic combination of lenalidomide, CPA, and dexamethasone. Reported median values of PFS and OS were 12,1 and 29.0 months, respectively [233]. A different phase 2 study evaluated low dose daily administration of pomalidomide and CPA in lenalidomide pretreated RRMM patients yielding an ORR of 67% and PFS of 14 months [234].

For patients who are not eligible for stem cell transplantation, adequacy of low dose thalidomide maintenance was assessed after standard induction chemotherapy. With 24 months of thalidomide maintenance, median PFS and OS were 27 and 39 months, respectively [235].

In summary, thalidomide and lenalidomide have proven efficacy with less pronounced toxicity in myeloma patients, which lead to their use widely, especially for maintenance in posttransplantation treatment.

### 3.13. Clinical Experience in Melanoma

Treatment of melanoma has shown a great advancement in the past decade with targeted therapies, immunotherapy, and combinations [236]. As angiogenesis plays a role in pathogenesis of melanoma and is known to be a prognostic factor, metronomic chemotherapy is a particularly appealing strategy [237]. Bhatt et al. used continuous infusion and paclitaxel 10 mg/m2 and oral celecoxib 400 mg twice daily in twenty patients. Median TTP was 57 days and OS was 212 days. Grade 3-4 toxicities were catheter-related only [238]. Another study used metronomic CPA for 3 weeks on 1 week off protocol for 13 unfit elderly people. Median OS was 8 months, ranging from 4 to 37 [239]. Ellebaek et al. combined CPA and COX inhibitor based metronomic therapy with immune modulatory autologous augmented dendritic cell (DC) vaccine. Metronomic CPA and a COX-2 inhibitor have been added to a DC vaccine with the intention to dampen. 8 patients had prolonged SD of 7-13 months [34]. TMZ was also retrospectively evaluated in a single-center experience; 33 patients were treated with cisplatin 75 mg/m^2^ every 28 days and TMSZ 75 mg/m^2^ for days 2-21 in patients with younger age and good performance; median PFS was 24 weeks and OS was 50 weeks [240].

As far as melanoma treatment and recent advances are concerned, still, main part of successful therapy lies on immunotherapeutic approaches and dual targeted therapies in driver mutation carrier patients. Besides, during the treatment process, temozolomide will definitely have a role.

### 3.14. Clinical Experience in Head and Neck Cancers

Advanced head and neck cancers are another group of cancer with limited surgical options and inadequate efficacy of cytotoxic chemotherapy.

Metronomic methotrexate and celecoxib were evaluated in platinum-resistant oral cancer without achieving an acceptable efficacy [241]. In another study in India, oral cancer evaluated the success of a metronomic regimen of oral methotrexate and celecoxib starting preoperatively and continuing as a maintenance after the standard treatment protocol. The disease-free survival rates were 86,5% in metronomic group versus 71.6% in control group, showing a statistical significance [242]. A similar study from India enrolling operable oral cavity cancer with maintenance metronomic therapy showed a median DFS of 13 months [243]. For head and neck cancer, another study evaluated metronomic oral regimen of methotrexate, erlotinib, and celecoxib in palliative treatment of patients with head and neck cancers and ineligible for MTD. Reported median PFS was 148 days [244]. Same authors retrospectively evaluated the adequacy of oral low dose chemotherapy for palliative treatment in a heterogenous group of head and neck cancer patients, revealing median OS of 155 days with oral cancers having a tendency for a shorter OS [245].

In the light of results of metronomic studies, it can be concluded that metronomic MTX (iv, weekly) may have a role in patients who are heavily treated and are still in need for chemotherapy for symptom control.

### 3.15. Clinical Experience in Miscellaneous Cancers

Metronomic chemotherapy was also experimented with other cancers.

Berruti et al. used long acting octreotide, metronomic capecitabine, and bevacizumab in metastatic well-moderately differentiated neuroendocrine tumors. The median PFS was 14.9 months. Biochemical response was seen in 52.9% and symptomatic response was seen in 82.3% of cases [246].

Metronomic chemotherapy is also a feasible instrument for treating sarcomas, as angiogenesis is a rational target to control the disease and the typical population bearing the cancer can possibly be debilitated for standard doxorubicin or ifosfamide based treatment, so administrating a more palliative and tolerable regimen is needed [247, 248]. Metronomic cyclophosphamide with daily prednisolone was administered to 26 elderly sarcoma patients with one-week cycle. It was reported that grade 3-4 lymphopenia was seen in 81% of patients. Total response rate was 26.9% [249].

## 4. Discussion

There is no doubt that metronomic chemotherapy has been a great enthusiasm. Medical society needs an innovative consensual intellect to seize the expanding knowledge about biology of cancer and deploy this knowledge to develop strategies to manage and treat cancer. Metronomic chemotherapy is a seminal model for this intellect, integrating the concepts of angiogenesis and angiogenetic machinery, tumoral microenvironment, cancer stem cells, and tumoral immunology. With this collaboration of molecular biological studies, for preclinical and clinical investigations together, an appreciable prospect for metronomic chemotherapy is still being constituted. Its befitting use with inherently advancing molecular-targeted molecules, especially the ones administered on daily basis such as tyrosine kinase inhibitors and mTOR inhibitors, is of a great potential [250]. Also the individualization of chemotherapy, that is, personalized medicine, is another role well suited for metronomic regimens [251]. Positioning the patient and the tumor at the center of the cancer management, it may be possible to tailor the administered drugs, doses, and schemes at the future; metronomic regimens are more feasible for this compared to maximum tolerated dose. Of course this tailoring process needs a feedback, namely, a surrogate marker to monitor the metronomic therapy. Although some were suggested, until now ideal predictive biomarker was to be agreed in the literature [252]. This is a drawback of metronomic therapy as its therapeutic activities are not appropriate for supervision. The cost of treating cancer is another matter in question. The economic burden of cancer treatment is increasing with more expensive drugs and increasing incidence [253]. Bocci et al. compared the outcomes and healthcare related costs of metronomic regimens versus novel treatments strategies in breast cancer. The study, which is the only pharmacoeconomic evaluation of MTC, nicely demonstrated the feasibility of MTC as being more cost-effective. Metronomic chemotherapy is advantageous over maximum tolerated dose, with lower doses, less parenteral administrations, and lower complication rates and thus lesser need for infrastructure [6].

For the past 15 years, metronomic treatment models have been comprehensively assessed for replacing, augmenting, or appending conventional regimens in miscellaneous cancers. Most published studies are preclinical, phase I and phase II. All-embracing quantitative data of clinical efficacy is reviewed elsewhere; it is possible to say that for the most they are fairly comparable to standard regimens [254]. But not to extrapolate the clinical data, phase III studies are needed. To date, there are a handful of phase III studies and there are 13 ongoing trials registered to the database of U.S. National Institutes of Health. More phase III studies are needed to establish the role of metronomic chemotherapy at the current and future cancer management. But, to quote Sir William Osler, “the value of experience is not in seeing much, but in seeing wisely”; there is no time to be lost to benefit the odds of metronomic regimens, but it still appears to be unfinished. The more reasonable strategy for now seems to be continuing investigations and step by step integrating the metronomic treatments to our current practice instead of sweeping it aside.

## Figures and Tables

**Table 1:** Table showing the studies using metronomic regimens in breast cancer.

Author	Treatment	N	Patient type	SD	PR	CR	ORR	TTP	CB	PFS	OS
Colleoni, 2002	CP 50 mg qd po MTX 2.5 mg bd 2 days of 1 week po	63	Metastatic pretreated	-	16%	3%	19.0%	-	31.7%	-	-

Colleoni, 2006	CP 50 mg qd po MTX 2.5 mg bd on 1st and 4th days po	86	Metastatic, pretreated and untreated	-	17%	3	20.9%	-	41.5%	-	-

Colleoni, 2006	CP 50 mg qd po MTX 2.5 mg bd on 1st and 4th days po Thalidomide 200 mg qd po	85	Metastatic, pretreated or untreated	-	8%	3	11.8%	-	41.5%	-	-

Orlando, 2006	MTX 2.5 mg bid on 1st and 2nd or 4th days po CP 50 mg qd po	153	Metastatic, pretreated or untreated	-	16%	5	-	-	15.7% (12 months )	-	-

Orlando, 2006	CP 50 mg qd po MTX 2.5 mg bid days 1, 4 q1w Trastuzumab 6 mg/kg q3w	22	Metastatic, pretreated, HER2 +	46%	18%		-	-	-	6 m	

Miscoria, 2012	CP 50 mg qd po MTX 2.5 mg bid days 1, 4 q1w	62	Metastatic pretreated		-	-	-		-	2.6m	7.1 m

Gebbia, 2012	CP 50 mg qd po	22	Metastatic pretreated hormone resistant	9%	3%	0%	-	3.8 m	?	-	12.8 m
	vs.	Vs.		Vs.	Vs.	Vs.		Vs.			Vs.
	CP 50 mg qd po with MTX 2.5 mg bid 2 days per week	39		12%	8%	0%		4.2 m			14 m
					

Wong, 2010	CP 50 mg qd po MTX 2.5 mg bid po two days q1w Prednisone 5 mg qd	41	Metastatic, pretreated or untreated	7%	2%	15%	24%	10 w	24%		48 w

Garcia-Saenz, 2008	CP 50 mg qd MTX 1 mg/kg iv q14d Bevacizumab 10 mg/kg iv q14d Trastuzumab (in HER2 +)	22	Metastatic, pretreated, HER2 +/-	32% (24 w)	32%	-	-	-	63.6%	7.5 m	13.6 m

Mayer, 2012	CP 50 mg qd MTX 2.5 mg qd 2 days per week Vandetanib 100 mg / 200 mg / 300 mg qd	20	Metastatic, pretreated or untreated	15% (24 w)	10%	-	-	-	-	-	-

Perroud, 2013	CP 50 mg qd po Celecoxib 200 mg bid po	155	!!	3% (24 w)	1 patient	-	-	14 w	46.7%	24 w (40%)	12 m (46.7%)

Aurilio, 2012	CP 50 mg qd po MTX 2.5 mg bd on 1st and 4th days per week po Fulvestrant 250 mg im q28d	32	Metastatic, pretreated, HR +	!!	!!	!!	!!	!!	56%	-	-

Crivellari, 2013	CP 50 mg qd po MTX 2.5 mg bd on 1st and 4th days per week po	36	Non-metastatic, >65 y, untreatable, HR-	-	Closed early	-	-	-	-	42 w (81%)	-

Dellapasque, 2011	CP 50 mg qd po Liposomal Doxorubicin 20mg/m2	29	Non-metastatic, untreatable, preoperatively	34.5%	62.1%	-	-	-	-	-	-

Soriano, 2011	CP 50 mg qd po MTX 2.5 mg bid 1E10-Alum	21	Metastatic,	4%	-	-	-	9.8 m	-	-	12.9 m

Dellapasque, 2008	CP 50 mg qd po Capecitabine 500 mg tid po Bevacizumab 10 mg/kg q2w	46	Metastatic	17%	46%	2%		42 w	68%	-	-

Licchetta, 2010	CP 50 mg day 1-21 q28d po Megestrol acetate 80 mg bid po	29	Metastatic, pretreated, HR +/-, HER2 +/-				31%	7.4 m			13.4 m

Wang, 2012	CP 65 mg /m2 iv days 1-14 q3w Capecitabine 1000 mg/m2 bid days 1-14 q3w	68	Metastatic, pretreated	-	-	-	30.3%	5.2 m	53.0%	-	16.9 m

Yoshimoto, 2012	CP 33 mg/m2 bid days 1-14 q3w Capecitabine 828 mg/m2 bid days 1-14 q3w	51	Metastatic, HER2- ER-/ER+	13%	-	-	44.4%	-	-	10.7/13.2	1 y (86%) 2 y (71%)

Smith, 2000	5-FU 1 mg/m2 days 1-28 q35d po Eniluracil 10 mg/m2 days 1-28 q35d po	29	Metastatic	24% (3 m)	55%						

Taguchi, 2010	Capecitabine 825 mg/m2 bid days 1-21 q 28d	33	Metastatic, untreated recurrent	24% (> 6 m)	-	-	18%	-	-	6.9 m	24.8 m

Fedele, 2012	Capecitabine 1500 mg qd	58	Metastatic, pretreated		2 (pretreated with Standard Capecitabine)	7 (pretreated with Standard Capecitabine)		7 m	62%		17 m

Watanabe, 2009	UFT 300 mg tid		T0, high risk, adjuvant	-	-	-	-	-	-	(RFS) 5 y (87.8 %)	5 y (96.2 %)

Cazzaniga, 2014	Capecitabine 500 mg tid Vinorelbine 20-30-40 mg/tot	31	Metastatic, pretreated,	-	-	-	-	-	58.1%	-	-

Young, 2012	Capecitabine 1250 mg/m2 qd Docetaxel 15mg/m2 Celecoxib 200 mg bid	38	Metastatic, pretreated	8% (6 m)	34%	-	-	3.6 m	42%	-	-

Schwartzberg, 2014	Capecitabine 1500/2000 mg daily in divided doses Fulvestrant 500 mg day 1, 250 mg day 1, 15, 28 followed by 250 mg q28d	41	Metastatic, HR+, HER2-	-	-	-	-	26.94 m	-	14.98 m	28.65 m

Otsuka, 2015	Irinotecan 60 mg/m2 days 1, 8, 15 q4w TS-1 80 mg/m2 days 3-7, 10-14, 17-21 q4w	34	Metastatic, recurrent	3%	44%	-	-		-		

Alagizy, 2015	Capecitabine 500 mg bid po	41	Operated, neoadjuvant FEC100+, +/- Postoperative RT, HR-, HER2-	-	-	-	-	-	-	-DFS- 42.4 m	44.34 m (estimated)

Addeo, 2012	Temozolomide with radiotherapy and following 4 w Temozolomide 75 mg/m2 days 1-21 q4w Vinorelbine 70 mg/m2 1,3,5 weekly for 3 w q4w, max 12 cycles	36	Untreated brain metastasis,		44%	8%	52%	-	-	8 m	11 m

Addeo, 2013	Vinorelbine 70 mg/m2 1,3,5 weekly for 3 w q4w, max 12 cycles	34	Metastatic	-	32%	6%	-	-	-	7.7 m	15.9 m

Saloustros, 2011	Vinorelbine 50 mg 3 times per week Bevacizumab 10 mg/kg 2 times per week q28d	13	Metastatic, pretreated	46%	8%	Closed early	-	-	-	-	-

De Iuliis, 2015	Vinorelbine 30 mg q2d	32	Metastatic		-	-	-	-	50%	-	-

Bottini, 2006	Letrozole 2.5 mg qd	57	HR+,		-	-	71.9%			-	-
	Letrozole 2.5 mg qd and CP 50 mg qd 6 months	57					87.7%				

Manso, 2013	NP-Liposomal Doxorubicin 30 mg iv 5-FU 500 mg iv Vincristine 0.25 mg iv CP 50 mg qd po Prednisone 20 mg	38	Metastatic, pretreated	27%	-	-		281 d		8.4 m	21 m

Masuda, 2014	Paclitaxel 80 mg/m2 days 1. 8. 15, 4 cycles Cyclophosphamide 50 mg qd po 4 cycles Capecitabine 1200 mg/m2 qd 4 cycles 5-FU 500 mg/m2 q3w, 4 cycles Epirubicin 100 mg/m2 q3w, 4 cycles CP 500 mg/m2 q3w, 4 cycles	33	HR-, ER-, preoperative	-	54.5%	-	-	-	31 (93.9%)	-	-

Montagna, 2012	Capecitabine 500 mg tid CP 50 mg qd Bevacizumab 15mg/kg q3w Erlotinib 100 mg qd	24	Metastatic, untreated, HR -, HER2-	21% (9 w)	58%	4%	-	-	75%	43 w	-

Munzone, 2010	P Liposomal-Doxorubicin	45	Metastatic, untreated, pretreated	39%	18%	-	-	-	45%	-	-

Mutlu, 2015	CP 50 mg qd po Etoposide 50 mg bid 2 days per week	-	Metastatic, pretreated			-	-	-		7.03 m	32.5 m

Taguchi, 2013	Capecitabine 828 mg/m2 bid po days 1-21 q28d Paclitaxel 80 mg/m2 days 1, 8, 15 q28d	43	Metastatic, pretreated				46.5%			8.3 m	22.9 m

Ambros, 2014	Capecitabine 1000 m2 bid po days 1-14 q21d	86	Metastatic, pretreated, HER2-				24.3%	7 m	55.8%		24.0 m

Neskovic, 1996	Etoposide 50 mg/m2 po days 1-14 q 28d	18	Metastatic, untreated, pretreated		28%	6%					

Yuan, 2015	Etoposide 60 mg/m2 po days 1-10 q 21d	75	Metastatic, pretreated	39%	9%					4.5 m	

**Table 2:** Table showing the studies using metronomic regimens in prostate cancer.

Author	Treatment	N	Patient type	PSA	Duration of Response	PR	TTP	PFS	OS
Bracarda, 2000	CP 2 mg/kg qd days 1-14 q28 d Estramustine 10 mg/kg/day days 1-14 q28d	32	Hormone refractory	43.7%	-	-	-	-	-

Pienta, 2001	Etoposide 50mg/m2 qd days 1-21 q28d Estramustine 15 mg/kg qd days 1-21 q28d	55	Hormone refractory	22%					

Nishimura, 2001	CP 100 mg qd UFT 400 mg qd Estramustine 560 mg qd	21	Hormone refractory	57%	7 m	-	-	-	-

Robles, 2003	Vinorelbine 25 mg/m2 iv q7d 12 weeks than q14d Prednisone 10 mg qd po	14	Hormone refractory, metastatic	36%					

Glode, 2003	CP 50 mg qd Dexamethasone 1m g qd	34	Hormone refractory	58%	8 m	-	-	-	-

Hellerstedt, 2003	CP 100 mg days 1-20 q30d Prednisone 10 mg qd DES 1 mg qd	36	Hormone refractory	42%	4.5 m	-	-	-	16.4 m

Lord, 2007	CP 50 mg qd	58	Hormone refractory	34.5%	7.5 m	-	-	-	-

Venkirataman, 2008	Dexamethasone 0.5 mg qd	102	Castration resistant	49%					

Fontana, 2009	CP 500 mg/ m^2^ bolus than 50 mg qd po Celecoxib 200 mg bid Dexamethasone 1 mg/day po	28	Hormone refractory	32%	-	-	-	3 m	21 m

Ladoire, 2010	CP 50 mg qd Prednisolone 10 mg qd	23	Hormone refractory	26%	-	-	-	6 m	11 m

Nelius, 2010	CP 50 mg qd Dexamethasone 1 mg/day po	17	Hormone refractory	23.5%	-	-	-	-	24 m

Gebbia, 2011	CP 50 mg qd MTX 2.4 mg po two times a week LHRH analogue	58	Castration resistant,	25%	24%	28%	-	-	-

Hatano, 2011	CP 100 mg qd UFT 400 mg qd Dexamethasone 10 mg/day	57	Hormone refractory	63%	-	-	13.3 m	-	-

Jellvert, 2011	CP 50 mg bid weeks 1,3,5 Ketoconazole 200 mg tid weeks 1,3,5 Etoposide 50 mg bid weeks 2,4,6 Estramustine 140 mg bid weeks 2,4,6	17	Castration resistant,	59%	-	-	-	-	-

Khan, 2011	CP 50 mg qd MTX 2.5 mg bid two times a week Celecoxib 400 mg bid	69	Hormone refractory	N/A			57 days		

Meng, 2012 ®	CP 50 mg qd Capecitabine 1000 mg bid Thalidomide 100 mg qd Prednisone 5 mg bid	28	Castration resistant,	35.7%	-	-	-	4.7 m	19.5 m

Orlandi, 2013	CP 50 mg qd Cetuximab 200 mg bid po Dexamethasone 1 mg qd po	43	Castration resistant,	32%	-	-	-	634CC 2.2 m 634CG/GG 6.25 m	-

Derosa, 2014	CP 50 mg qd po Docetaxel 06 mg/ m^2^ in q21d Prednisone 10 mg qd from day 2 Celecoxib 400 mg qd	41	Castration resistant, untreated	82%	-	-	-	-	-

Yashi, 2014	CP 50 mg qd po Dexamethasone 1 mg qd po	24	Castration resistant, metastatic	33.3%	-	-	-	5.0 m	19.0 m

Zhu, 2014	Etoposide 25mg/m2 bid days 1-21, q28d Prednisone 5 mg bid days 1-21, q28d	39	Castration resistant,	41%				5.9 m	

Barroso-Sousa, 2015 ®	CP 50 mg qd or CP 150 mg days 1-14, q21d Prednisone 10 mg po	40	Castration resistant, pretreated, metastatic	20%	-	-	-	-	-

Wang, 2015	CP 50 mg qd po Lenalidomide 25 mg qd days 1-21 q28d	6	Castration resistant, metastatic	31.7%	-	-	-	-	-

Petrioli, 2015	Abiraterone 25 mg qd po Prednisone 5 mg qd po	26	Castration resistant	69.2%				6.4 m	14.3 m

**Table 3:** Table showing the studies using metronomic regimens in ovarian cancer. Bev, Bevacizumab; CP, Cyclophosphamide.

Author	Treatment	N	Patient type	SD	PR	CR	ORR	TTP	CB	PFS	OS
Chura, 2007	Bev 10 mg/kg q14d CP 50 mg qd po	15	Pretreated, recurrent	3 (20%)	6 (40%)	2 (13.3%)	53%	-	-	-	-

Gordinier, 2007	Standard regimens	18	Pretreated, ovarian or primary peritoneal	60%	6.7%	-	-	-	-	3.7 m	-
	Thalidomide 200 mg qd	18		53.8%	5%					3.8 m	
	None	4		?	?					?	

Downs, 2008	Topotecan 1.25 mg/ m^2^days 1-5, q21d	30	Recurrent, epithelial, platinum refractory	-	17%	30%	47%	-	-	-	19 m
	Thalidomide 200 mg qd increasing to maximum tolerated Vs.	Vs.			Vs.	Vs.	Vs.				Vs.
	Topotecan 1.25 mg/ m^2^ days 1-5, q21d	39		-	3%	18%	21%	-	-	-	15 m

Garcia, 2008	Bev 10 mg/kg q14d CP 50 mg qd po	17	Recurrent, platinum refractory	-	17 (24%)	-	-	7.2 m	-	6 m (56%)	16.9 m

Jurado, 2008	Bev 10 mg/kg q14d CP 50 mg qd po	9	Recurrent, platinum refractory	2 (22%)	2 (22%)	2 (22%)	4 (44%)	5.5 m	-	6 m (33%)	-

Hurteau, 2010	Thalidomide 200 mg qd increasing to maximum tolerated or 400 mg vs Tamoxifen 20 mg qd po for 1 year	138	Stage III/IV, disease free after 1st line,	Closed early, interim analysis showed thalidomide was inferior to tamoxifen							

Sanchez-Munoz, 2010	Bev 10 mg/kg q14d CP 50 mg qd po	38	Pretreated, recurrent	3 (8.1%) (6 w)	12 (32.4%)	3 (8.1%)	-	-	-	4.5 m	10.7 m

Legge, 2011	Carboplatin AUC5, q28d Celecoxib 400 mg qd	45	Pretreated, recurrent		10	3	29%	-	-	5 m	13 m

McGonigle, 2011	Bev 10 mg/kg days 1, 13 q28d Topotecan 4 mg/ m^2^ days 1, 8, 15 q28d	40	Platinum resistant, ovarian / peritoneal / fallopian	14 (35%)	10 (25%)	-	-	-	-	7.8 m	16.6 m

Ramasubbaiah, 2011	Sorafenib 400 mg qd Topotecan 3.5 mg/ m^2^ days 1, 8, 15 q28d	14	Platinum resistant,	14 (46.7%)	5 (16.7%)	-	-	-	-	-	-

Kucukoner, 2012	Etoposide 50 mg qd po days 1-14 q28d	51	Platinum resistant,	25.5%	17.6%					3.9 m	16.4 m

Barber, 2013	Bev 10 mg/kg q14d CP 50 mg qd po	66	Pretreated, recurrent	15 (22.7%)	21 (31.8%)	7 (10.6%)	42.4%			5 m responders)	20 m responders)

Ferrandina, 2014 ®	CP 50 mg qd po	54	Platinum resistant/sensitive	-	11 (20.4%)	-	20.4%			4 m	13 m

Bhattacharyya, 2015	CP 50 mg qd po TMZ 20 mg bid days 1-14, q21d	55	Platinum resistant,		24	N/A	44%			5.9 m	10.1 m

Roque, 2015 ®	Ixabepilone 16-20 mg/ m^2^ days 1, 8, 15, q28d	8/3	Uterine/Ovarian-fallopian-peritoneal	-	-	-	41.7%	-	-	3.0 m/-	-
	Vs										9.6 m
	Ixabepilone 16-20 mg/ m^2^ days 1, 8, 15, q28d Bev 10 mg/kg q14d	16/33		-	-	-	-	-	-	6.5 m/ -	-

**Table 4:** Table showing the studies using metronomic regimens in hepatocellular cancer. UFT, Tegafur-5 FU.

Author	Treatment	N	Patient type	SD	PR	CR	ORR	TTP	PFS	OS
Hsu, 2010	Sorafenib 400 mg bid UFT 125mg/m2	53	Untreated, Child-Pugh class A	26 (49%)	4 (8%)				3.7 m	7.4 m

Boige, 2012	Bevacizumab 5 mg/kg or 10 mg/kg q14d	43	Advanced	N/A	6 (14.0%)	N/A	14.0%			

Shao, 2012	Thalidomide 100 mg bid UFT 125mg/m2	43	Untreated,				9%		1.9 m	

Woo, 2012	(Into the hepatic artery) Epirubicin 30 mg/m2 q28d Cisplatin 15mg/m2 q21d 5-FU 50 mg/m2 q21d	30	Portal vein thrombosis		6 (20.0%)			63 d		63 d

Brandi, 2013	Capecitabine 500 mg bid	59	Untreated	30	1	2			6.03 m	14.47 m
		31	Sorafenib refractory	10	N/A	N/A			3.27 m	9.77 m

Granito, 2015	Capecitabine 500 mg bid	26	Sorafenib refractory					4 m		8 m

**Table 5:** Table showing the studies using metronomic regimens in glioblastoma multiforme (GBM). CP, Cyclophosphamide; TMZ, Temozolomide.

Author	Treatment	N	Patient type	SD	PR	ORR	TTP	PFS 6	OS
Brandes, 2006	TMZ 75 mg/ m^2^ days 1-21 q28d	33	Chemonaive, refractory to RT and/or surgery	-	-	9%	-	30.3%	-

Kesari, 2007	Etoposide 35 mg/ m^2^ days 1-21 CP 2 mg/kg days 22-42, Thalidomide Celecoxib	48	GBM and AG	59%	11%	-	-	11 w (GBM) 14 w (AG)	41.5 w (GBM) 42 w (AG)

Balmaceda, 2008	TMZ 200 mg/ m^2^ followed by 90 mg/m2 q12h 9 times	120	GBM, anaplastic astrocytoma, anaplastic oligodendroglioma	-	-	-	-	4.2 m 5.8 m 7.7 m	8.8 m 14.6 m 18 m

		1:21	1:GBM-refractory to conventional TMZ,					1:17%	
Perry, 2008	TMZ 50 mg/ m^2^ qd	2:14	2:GBM-refractory to conventional and adjuvant TMZ	-	-	-	-	2:57%	-
		3:14	3:AG-refractory to conventional TMZ,					3:42%	

Clarke, 2009	TMZ 50 mg/ m^2^ qd	43	Pretreated with standard TMZ + RT	-	-	-	-	-	15.1 m

Reardon, 2009	Bevacizumab 10 mg/kg two times a week	59	GBM and grade 3 glioma	-	-	-	-	40.6%	63.1 w
	Etoposide 50 mg/ m^2^ qd days 1-21 q30d							44.4%	44.4 w

Kong, 2010	TMZ 40 mg/ m^2^ m^2^ qd or 50 mg/ m^2^qd	38	Pretreated GBM	-	-	-	-	32.5%	56.0% (6 m)

Stockhammer, 2010	TMZ 10 mg/ m^2^bid Celecoxib 200 mg	28	Pretreated GBM	-	-	-	4.2 m	43 %	-

Verhoeff, 2010	Bevacizumab 10 mg/kg q21d TMZ 50 mg/ m^2^ qd	23	High grade glioma	-	-	20%	-	17.4 %	17.1 w

Reardon, 2011	Bevacizumab 10 mg/kg q14d With Etoposide 50 mg/ m^2^ days 1-21 q 30d or With TMZ 50 mg/ m^2^ m^2^ qd	23	Bevacizumab pretreated GBM,	52%	-	-	-	4.4%	-

Omuro, 2013	TMZ 50 mg/ m^2^ qd	47	Pretreated grade 3 malignant glioma and GBM	-	-	-	-	19%	7 m

Zustovich, 2013	TMZ 40 mg/ m^2^qd Sorafenib 400 mq qd	43	Pretreated GBM	-	-	-	-	26%	7.5 m
